# Methanol intoxication “Eau de vie” in Morocco from 2013 to 2020

**DOI:** 10.1192/j.eurpsy.2023.1398

**Published:** 2023-07-19

**Authors:** O. Seyar, L. Azizi, M. Sabir, F. El Omari, H. Chaoui, N. Rhalem, R. Soulaymani Bencheikh

**Affiliations:** 1 psychiatrics, University Hospital Arrazi, Salé; 2 Toxicology, Anti-Poison and Pharmacovigilence Centre, Rabat, Morocco

## Abstract

**Introduction:**

Methanol intoxication is a public health problem in developing countries and can be ingested accidentally or with suicidal intent, leading to intoxication in isolated or collective forms. Methanol is used as a substitute for ethyl alcohol in several adulterated alcoholic beverages such as “eau de vie”, which is a drink distilled from dried fruits, such as dates, grapes and figs. Inside the body, it is metabolised into formic acid which, if left untreated, affects brain tissue, leads to blindness and can also cause death.

**Objectives:**

The objective of this retrospective study of a series of cases was to describe the epidemiological characteristics of methanol “eau de vie” poisoning cases collected by the Anti-Poison and Pharmacovigilance Centre of Morocco between 2013 and 2020 and to explain these results.

**Methods:**

This is a descriptive and retrospective cross-sectional study over a period of 7 years from 1 January 2013 to 31 December 2020, which concerned 16 cases of intoxication by methanol “eau de vie” reported to the Anti-Poison and Pharmacovigilance Centre of Morocco, the study population concerned the entire Moroccan population throughout the territory of Morocco. The analysis concerned the frequency, the distribution in time, the distribution in space, the characteristics of the intoxicated, the type and circumstances of the intoxication and its evolution.

**Results:**

The CAPM recorded, during the study period, 16 cases of intoxication by methanol “Eau de vie” in Morocco. These cases were reported by telephone in 93.75% of the cases and collected by studies on hospital registers in 6.25% of the cases. Men were more affected than women. The most affected age group was adults, accounting for 50%. Adolescents accounted for 37% of cases and children for 13%. Drug addiction was the most frequent circumstance, followed by accidental intoxication and voluntary intoxication. The most frequently encountered signs were gastrointestinal signs followed by central and peripheral nervous system signs and heart rate and rhythm disorders. The outcome was favourable in 62% of cases, 6% with blindness after-effects and death occurred in 19% of cases.

**Conclusions:**

Methanol poisoning can result from the consumption of illegal products containing methanol such as brandy, hence the importance of raising public awareness of this danger. It is also necessary to make health professionals aware of the clinical signs of methanol poisoning and what to do in the event of intoxication.

**Disclosure of Interest:**

None Declared

